# Socio-economic inequalities in the financing of cardiovascular & diabetes inpatient treatment in India

**Published:** 2011-01

**Authors:** Krishna D. Rao, Aarushi Bhatnagar, Adrianna Murphy

**Affiliations:** Public Health Foundation of India, New Delhi, India; * *Harvard School of Public Health, Boston, MA, USA*

**Keywords:** CVD, diabetes, out-of-pocket payment, self-reported prevalence, socio-economic

## Abstract

**Background & objectives:**

Cardiovascular disease (CVD) and diabetes have become a leading threat to public health in India. This study examines socio-economic differences in self-reported morbidity due to CVD and diabetes, where people having these conditions seek care, how much households pay for and how they finance hospital treatment for these conditions.

**Methods:**

Data for this study are taken from the National Sample Survey Organization (NSSO) 60^th^ round on ‘Morbidity and Health Care’ conducted between January and June 2004. Information from 2,129 and 438 individuals hospitalized for CVD and diabetes was analyzed.

**Results:**

The self-reported prevalence among adults was 12 per cent for CVD, 4 per cent (7% urban and 3% rural) for heart disease and 6 per cent (10% in urban and 4% in rural) for diabetes. Both self-reported CVD and diabetes appeared to afflict the wealthier more. The private sector was the main provider of outpatient and inpatient care for CVD and diabetes treatment, though the poor depended more on the public sector. Out-of-pocket payments (OOPS) for hospital treatment claimed a large share of annual household expenditures; 30 per cent for CVD and 17 per cent for diabetes. The OOPS share for diabetes treatment declined with increasing income. The majority of OOPS for hospital treatment paid by the poor was financed through borrowings.

**Interpretation & conclusions:**

The considerable financial strain which households, particularly the poor, face in treating CVD and diabetes is alarming. As the burden due to CVD and diabetes increases in India, more households will be subject to these financial strains and unfortunately, the economically vulnerable among them will be the worst affected. While primary prevention of these conditions need more emphasis, in addition, insurance schemes targeted at the poor like the RSBY have an important role to play in financially protecting vulnerable households.

Cardiovascular disease (CVD) and diabetes have become a leading threat to public health worldwide. Contrary to popular assumption however, these commonly titled “diseases of affluence” are neither restricted to the developed world nor to the wealthier within countries. In fact, the growing dominance of chronic diseases in the share of global disease burden is due to their increase in low/middle income economies, especially India, China and Eastern Europe^1^. In India, the prevalence of coronary heart disease; in urban areas prevalence has increased from about 2 per cent in 1960 to 6.5 per cent in 1970, 7.0 per cent in 1980, 9.7 per cent in 1990 and 10.5 per cent in 2000 and in rural areas it increased from 2 per cent in 1970 to 2.5 per cent in 1980, 4 per cent in 1990 and 4.5 per cent in 2000^2^. The rising prevalence of chronic diseases in low/middle income nations has been attributed to dietary factors, high rates of smoking and alcohol, and increasingly sedentary lifestyles. Available evidence from developed countries indicates that the burden of chronic diseases and their risk factors are predominantly concentrated among the economically poor^3^,^4^. While there is little available evidence of the socio-economic distribution of chronic diseases in low/middle income countries, the poor appear to be disproportionately burdened with the risk factors associated with these diseases. For example, smoking prevalence, which is a risk factor for a variety of chronic diseases including CVD, is higher among the poor in low/middle income countries^4^. The prevalence of obesity among women also appears to be shifting to those in the lower economic groups^5^.

Some studies which have examined socio-economic differences of CVD and diabetes prevalence in India have been based on small populations due to the lack of reliable cause of death registries or nationally representative data. Further, no clear pattern in the socio-economic gradient, particularly for CVD, emerges from their findings. A study on a semi-urban population in southern India found that higher socio-economic status was associated with greater prevalence of CHD risk factors^6^. In contrast, a study of industrial workers found that risk factors (tobacco use and hypertension) for CVD were concentrated among the lesser educated in both urban and rural areas, however, the prevalence of diabetes and being overweight increased with better education^7^. In rural northern India the prevalence of clinically diagnosed coronary heart disease and risk factors (smoking and hypertension) were higher among lesser educated groups^8^. Further, findings from epidemiological studies suggest that prevalence of coronary heart disease increases from rural to semi-rural to urban areas^2^. Recent national surveys indicate that the prevalence of self-reported diabetes among adults (15 to 49 yr of age) increases with economic status while tobacco consumption decreases with better education and economic status^9^.

The treatment of chronic diseases like CVD and diabetes is expensive and can consume a substantial part of a household’s financial resources since the patients require treatment over a long period of time and often require hospitalization. In countries like India where health care is predominantly financed through out-of-pocket payments, financing treatment of chronic diseases can be particularly burdensome, especially for poorer households. One study of diabetes patients at a private hospital in south India estimated that medical costs amounted to between 15 and 25 per cent of household income^10^. To accommodate unexpected and high health care spending, households either reduce the consumption of other goods and services leaving them more vulnerable to impoverishment, forego treatment, borrow money, or sell assets. Chronic diseases also negatively impact the economic well being of households when they claim productive household members; one-third of CVD deaths occur in the working and child rearing age populations^11^. Further, people with chronic conditions have been found to have lower productivity^12^. Early mortality and disability in this age group can severely affect a household’s financial well being. This study explores the socio-economic inequalities in the care-seeking and financing of CVD and diabetes hospitalization. Specifically, it examines socio-economic differences in self-reported morbidity due to CVD and diabetes, where people having these conditions seek care, how much households pay and the different sources used to finance treatment. While earlier research on this issue has been based on small populations, this study uses data from a nationally representative household survey. Further, the issue of socio-economic inequalities in out-of-pocket expenditures and methods of financing treatment of chronic diseases like CVD and diabetes has not received the attention it deserves in India.

## Material & Methods

Data for this study are from the National Sample Survey Organization (NSSO) 61^st^ round on ‘Morbidity and Health Care’ conducted between January and June 2004^9^. The NSSO 61^st^ round is a multi-staged cluster sample survey covering all 35 States and union territories in India. In the first stage, census villages in rural and wards in urban areas were sampled. In the second stage, households were sampled from each selected village or ward. The total of 47,302 rural and 26,566 urban households were surveyed. Each sampled household was administered a standardized questionnaire. The survey made efforts to interview each adult member of the household. Information on children in the household were collected from their mothers. For the purposes of this study, outpatients and inpatients were analyzed separately because of the differing time periods for which information on them was collected. The study sample for individuals reporting diabetes ailments included a total of 1,420 outpatients and 438 inpatients. For CVD, the study sample included 3,181 outpatients and 2,129 inpatients.

The survey collected information on the morbidity and various aspects of household care seeking such as outpatient and inpatient use of health care services provided by the public and private sector, health expenditures incurred and the means employed by households to finance health care. Detailed information was collected on health expenditures for hospitalized cases, including, doctors fees, medicines, diagnostics, bed charges, attendant charges, physiotherapy, medical appliances, others (food, blood, oxygen, ambulance), transport of household members and their food and lodging. Further, reimbursement received for these expenditures was recorded. In addition, information on household consumption expenditure and asset ownership was also collected.

*Identifying CVD and diabetes cases*: The NSSO 61^st^ round asked about the ailment spells experienced by each household member in the 15 days before the survey. A person was treated as ailing if he/she was under medication for an ailment during the reference period, whether he/she felt sick or not. Further, each person was probed about the signs/symptoms to better ascertain their particular ailment.

All spells of ailment (an ailment spell being defined as a continuous period of sickness owing to a specific ailment) suffered by each household member, both present as well as the deceased, during this period were recorded. The nature of the self-reported ailment was coded with reference to a list of ailments available with the interviewer. This list included diabetes mellitus and CVD; within CVD two sub-categories were identified - heart disease and hypertension. It is important to note that these are not medically diagnosed conditions (though these may be) but self-reported conditions. Use of health services for each ailment spell and details of outpatient visits made for each ailment spell in the past 15 days prior to the survey were also collected. For hospitalized treatment, information was collected for every event of hospitalization of each household member, whether living or deceased at the time of survey, during the 365 days preceding the date of the survey. The health expenditure and financing analysis was limited to hospitalizations.

From the study sample various outcomes of interest were estimated. These included the self-reported prevalence of CVD and diabetes, proportion of ailment spells medically treated (though classification by the qualification of the provider was not included), average out-of-pocket spending (OOPS) on diabetes and CVD hospital treatment, share of expenditures on diabetes and CVD hospital treatment in annual household consumption expenditure and the proportion of households using different methods of financing expenditures on CVD and diabetes treatment. Estimates of treatment expenditures were adjusted for insurance reimbursements.

To examine the distribution of these outcomes of interest across economic groups, per capita expenditure cut-offs based on the population distribution of per capita household expenditure were calculated to divide the population into three economic groups – poorest 40 per cent, middle 40 per cent and richest 20 per cent. The usual method of disaggregating the population into quintiles was not used as the sample size of inpatients within each quintile was very small. Individuals in the study sample were then placed in one of these three groups based on their per capita household consumption expenditure. The outcomes of interest were then estimated for each group. All estimates were weighted. There were very few missing observations in the study sample and so no imputation was carried out.

## Results

Table I describes the socio-demographic characteristics of the study sample of individuals who were hospitalized in the past year for CVD and diabetes treatment. Patients were middle age on average and included males and females in equal proportions. The majority patients were married, had some formal education and tended to be from rural areas.

**Table I:** Sample characteristics of inpatients

Characteristic	CVD	Diabetes
Sample (number of individuals)	2, 129	438
Age (yr)	52 ± 36.60	55 ± 21.61
Male (%)	54	51
Married (%)	77	77
Urban (%)	46	42
Education (%)		
No schooling/illiterate	34	27
Primary or below	26	37
Middle and higher schooling	31	29
Diploma/graduate & above	9	7
	100	100
Monthly per capita consumption expenditure ( 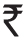 )	926 (649.53)	972 (653.54)

Table II describes the distribution of self-reported prevalence and care-seeking for CVD and diabetes across income groups. Individuals in the poorest 40 per cent of the population were from households with a monthly per capita consumption expenditure (MPCE) of 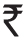 420 or below, those in the middle 40 per cent have MPCE between 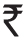 420 and 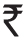 760, and those in the richest 20 per cent have MPCE of 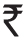 760 and above. India’s official poverty line is 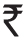 356 in rural and 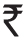 539 in urban areas per month per person and reflects the minimum amount needed to purchase 2200 calories per day^12^. The poorest group will, therefore, contain all rural and most urban individuals below the poverty line. Further, given that the poverty line reflects the minimum nutritional requirements, individuals in the middle 40 per cent group will also be economically vulnerable though they are above the poverty line.

**Table II:** Self-reported prevalence and care-seeking for cardiovascular disease (CVD) and diabetes among adults (≥ 20 yr)

	CVD				Diabetes			
	Poorest 40%	Middle 40%	Richest 20%	All	Poorest 40%	Middle 40%	Richest 20%	All
Had ailment in past 15 days (%)	5	9	22	12*	2	4	11	6*
Heart disease (%)	2	3	7	4*	-	-	-	-
Hypertension (%)	3	6	15	8*	-	-	-	-
Ailment spells medically treated (%)	90	95	98	96	97	96	98	97
Ailment spells treated in the public sector (%)	28	26	20	23*	27	22	16	19*
Hospitalization episodes in the public sector (%)	47	37	29	35*	52	35	24	31*
Sample (spells of ailment)	393	1,255	2,141	3,789	134	457	1,100	1,691

* Global ChiSq-test. *P* < 0.05

Overall, 12 per cent (8% in rural and 21% in urban areas) of individuals 20 yr and older reported having CVD and 6 per cent (4% in rural and 10% in urban areas) reported having diabetes in the 15 days prior to the survey (Table II). Among those reporting a CVD ailment, 4 per cent (3% rural and 7% in urban areas) had heart disease and 8 per cent (6% in rural and 14% in urban areas) had hypertension. The self-reported prevalence of CVD and diabetes is significantly different (*P* < 0.05) between the three income groups and increases with rising economic status.

Most of the CVD (96%) and diabetes (97%) ailment spells experienced by individuals were medically treated. In the case of CVDs, the proportion of treated ailment spells increased with rising economic status while for diabetes there was no discernable trend. Most of the CVD (77%) and diabetes (81%) ailment spells were treated in the private sector. Across all economic groups, the majority of ailment spells were treated in the private sector, though the proportion of ailment spells treated in the private sector increased with economic status. Most hospitalization episodes in the past year for CVD (65%) and diabetes (69%) were also treated in the private sector. However, a remarkable difference was found in where the poor seek hospital care compared to the rich; in the poorest group, 47 per cent of the hospitalization episodes for CVD and 52 per cent for diabetes were in public sector hospitals compared to 29 per cent for CVD and 24 per cent for diabetes among the richest group.

On an average, patients paid Rs. 12,317 (CVD) and 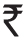 5925 (diabetes) out-of-pocket per hospitalization episode (Table III). The average amount increased with economic status. The average OPP per hospitalization episode was 3 times higher for CVD and 2 times higher for diabetes in the richest compared to the poorest group.

**Table III:** Out-of-pocket payments (OPP) in 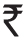 for CVD and diabetes hospitalization

Indicator	Poorest 40%	Middle 40%	Richest 20%	All
CVD				
Mean OPP payment per hospitalization	5,568	9,203	17,431	12,317*
OPP share of total annual household expenditure (%)	25	27	31	30*
Sample	393	906	1,152	2,451
Diabetes			
Mean OPP payment per hospitalization	4,152	5,106	6,959	5,925*
OPP share of annual household expenditure (%)	25	19	15	17*
Sample	63	210	270	543

Note: OPP are calculated for patients who reported paying something for their treatment. The sample size is the number of hospitalization episodes in which some OPP expenditure was incurred.

* Global F-test. *P*<0.05

Overall, the OOPS share of annual household consumption expenditure for CVD and diabetes hospitalization was 30 and 17 per cent respectively. This share increased with economic status for CVD patients but the opposite trend was seen for diabetes patients. Further, OPP for CVD and diabetes hospitalization consumed 25 per cent of the household consumption expenditure of poor patients.

Households used a variety of methods to finance OPP for hospital treatment. These included, among others, current income and savings, borrowing (from moneylenders, banks, friends/relatives) and in extreme situations, sale of their assets. The majority (57%) of total OOPS expenditures incurred by the households for CVD related hospitalizations were paid from household savings, followed by borrowings (35%) and the sale of assets (8%). For the poorest, only 38 per cent of OOPS expenditures were financed through savings which increased to 62 per cent for the richest group (Fig.). Infact, the majority of OOPS expenditures in the poorest group (55%) was financed through borrowings, the share of which declined as economic status increased. The share of asset sales as a financing source was similar across different economic groups.

**Fig.**
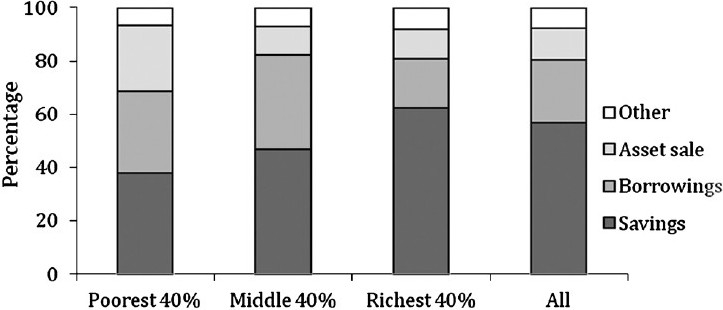
Sources of financing hospitalization for cardiovascular disease.

## Discussion

In India, the absence of a well-functioning disease surveillance system and reliable cause of death registries has limited measuring the national prevalence of CVD and diabetes. We used a nationally representative household survey to provide estimates of self-reported prevalence of CVD and diabetes and associated care seeking and out-of-pocket spending on hospital treatment. The disease conditions recorded in the survey were not clinically diagnosed cases but self-reported conditions. Consequently, these may not represent the ‘true’ prevalence of CVD and diabetes. Importantly, this bias can be larger among poorer economic groups which further affects the observed socio-economic differences in health expenditures^13^. However, because nearly all individuals reporting these ailments had sought medical care and the expenditure analysis was limited to hospitalization episodes, which are salient events, the extent of this self-reported bias may not be large.

In addition to the self-reporting bias, another limitation of the survey data was that it did not include asymptomatic patients among those who were ailing, thereby leading to the problem of undercounting. Also, the number of observations at the State level analysis became very small for specific ailments like diabetes or CVD which prevented s0 tate level analysis. The national level estimates tend to be based on a thin sample, particularly when disaggregated by socio-economic groups.

Findings from this study indicated that self-reported prevalence among adults in 2004 was 12 per cent for CVD, 4 per cent (7% urban and 3% rural) for heart disease and 6 per cent (10% in urban and 4% in rural) for diabetes. In comparison with previous studies, the reported prevalence of CVD and heart disease was lower and that of diabetes higher^2^,^14^–^17^. Both CVD and diabetes appear to afflict the wealthier more; the self-reported prevalence for both these conditions increased with higher income. While the observed socio-economic gradient makes it tempting to describe CVD and diabetes as diseases of affluence in India, there are several reasons to be cautious about coming to this conclusion. The lower prevalence of CVD and diabetes in the poorer economic groups might be because relatively fewer individuals in these groups reported having these conditions. One reason for this is individuals in these groups are unaware of their true condition due to a lack of access to medical care, diagnosis and testing. Secondly, those in the poorer economic groups are less likely to be able to identify conditions like heart disease, hypertension, and diabetes than those who are economically better-off and better educated. Finally, some of the risk factors for CVD like smoking are concentrated among the poorer economic groups.

Overall, both CVD and diabetes patients had remarkably good access to medical care, though the quality of this care is likely to be variable. The private sector was the main provider of outpatient and inpatient care for CVD and diabetes treatment. However, the poor depended more on the public sector. While most poor patients preferred the private sector for outpatient treatment of CVD and diabetes, for hospitalizations, nearly half the poor CVD and the majority of poor diabetes inpatients were treated in the public sector. This highlights the importance of the public sector in providing care to the poor.

Overall, OOPS for CVD and diabetes hospitalization consumed a substantial 30 and 17 per cent of household consumption expenditure respectively. The wealthier spent more on hospitalization compared to the poor and this difference was particularly large for CVD treatment. This reflects both where treatment is sought (*i.e*. public or private hospitals) and the procedures undertaken. The share of OOPS in household consumption expenditure increased with economic status for CVD and declined for diabetes hospitalization (*i.e*. regressive). For the poorest group, OOPS spending on CVD and diabetes treatment consumed 25 per cent of household consumption expenditure. Such a high share of consumption expenditure devoted to treatment by households which spend just enough to feed them themselves highlights the grave economic consequences they face from chronic diseases like CVD and diabetes.

More than 50 per cent of OOPS for CVD and diabetes treatment was paid through household savings. The poorer economic groups financed their OPP for hospitalization primarily through borrowings, (including money lenders, banks and friends/relatives), indicating the severe financial strain that these expenditures place on economically vulnerable households. The sale of assets did not appear to be concentrated in any particular economic group.

In conclusion, the findings from this study highlight the considerable financial strain which households, particularly the poor, face in treating CVD and diabetes. In the absence of access to affordable health care or insurance, vulnerable households resort to borrowing so that they may treat their sick. As the burden due to CVD and diabetes increases, more households will be subject to these financial strains and unfortunately, the economically vulnerable among them will be the worst affected. This highlights the critical need for policies to mitigate these adverse effects either through increasing the coverage of quality health services through the public sector or providing a viable insurance cover to households. Recent developments like the roll out of national insurance schemes (*e.g*. RSBY) for the poor are a step in the right direction because these cover hospitalization costs and can lower financial barriers to access. Equally important and in conjunction with these efforts, primary prevention strategies to reduce the burden of CVD and diabetes in the population need to be pursued.
